# Single-cell transcriptome analysis of muscle and blood tissues from a patient with a rare homozygous *ANO5* variant causing limb-girdle muscular dystrophy type R12

**DOI:** 10.3389/fgene.2026.1833196

**Published:** 2026-10-19

**Authors:** Ming Chen, Ge Dong, Yue Wang, Jingjing Liu, Zhiqin Wang, Dengyi Bao, Zhiqiang Huang, Xiaowen Li, Yanmei Li, Zhigang Cai, Wei Wei

**Affiliations:** 1 Department of Rheumatology and Immunology, Tianjin Medical University General Hospital, Tianjin, China; 2 Tianjin Medical University General Hospital Medical Center for Rheumatology and Immunology, Tianjin, China; 3 Department of Pharmacology, State Key Laboratory of Experimental Hematology, Tianjin Key Laboratory of Inflammatory Biology, School of Basic Medical Science, Tianjin Medical University, Tianjin, China; 4 The Province and Ministry Co-sponsored Collaborative Innovation Center for Medical Epigenetics; Department of Pharmacology, School of Basic Medicine, Tianjin Medical University, Tianjin, China; 5 Department of Bioinformatics, School of Basic Medical Science, Tianjin Medical University, Tianjin, China; 6 Department of Neurology, Tianjin Medical University General Hospital, Tianjin, China; 7 Department of Hematology, Tianjin Medical University General Hospital, Tianjin, China

**Keywords:** ANO5, limb-girdle muscular dystrophy type R12, muscle fibers, MYH7, single-cell RNA sequencing

## Abstract

**Background:**

Limb-girdle muscular dystrophy type R12 (LGMD R12) is a recessive genetic myopathy, typically caused by pathogenic variants in *anoctamin-5 (ANO5)*, which encodes a multipass membrane protein localized to the sarcolemma and sarcoplasmic reticulum of muscle fibers. Effective therapeutic strategies for LGMD R12 remain elusive. A comprehensive analytical framework is required to dissect the cellular and molecular changes underlying this disease.

**Methods:**

The *ANO5* variant in the patient and his family members was validated by Sanger sequencing. We performed single-cell RNA sequencing analysis on biceps brachii muscle tissue (MUS) and peripheral blood mononuclear cells (PBMCs) from healthy controls (HCs) and a patient who was homozygous for the pathogenic variant in exon 10 of *ANO5* (c.898dupA, p.Ile300fs). Immunofluorescence staining was conducted to validate key findings from the single-cell transcriptome analysis.

**Results:**

In the MUS cell atlas , we observed a significant loss of type I muscle fibers in the patient with LGMD R12. Accordingly, immunofluorescence staining revealed a dramatic loss of MYH7^+^ cells (MYH7^+^, a marker of type I muscle fibers) compared with those in controls. Glycolytic pathway activity and cellular metabolic activity were significantly downregulated, suggesting that they are two transcriptomic signatures in the muscle tissue of this patient. In addition, muscle progenitor cells from the patient exhibited impaired differentiation capacity, failing to generate mature myocytes. In both MUS and PBMCs, we also observed heightened immune crosstalk accompanied by significant activation of pathways such as LGALS9–CD45/CD44 and ANXA1–FPR1, suggesting immunological activation in this patient.

**Conclusion:**

LGMD R12 with pathogenic *ANO5* variants is a rare genetic disease. Our transcriptome and experimental analyses revealed dramatic cellular and molecular changes in affected tissue at the single-cell resolution in a patient with LGMD R12. This study provides valuable insights into the underlying mechanisms and potential development of interventions for this recessive genetic myopathy.

## Graphical Abstract

**Figure:**
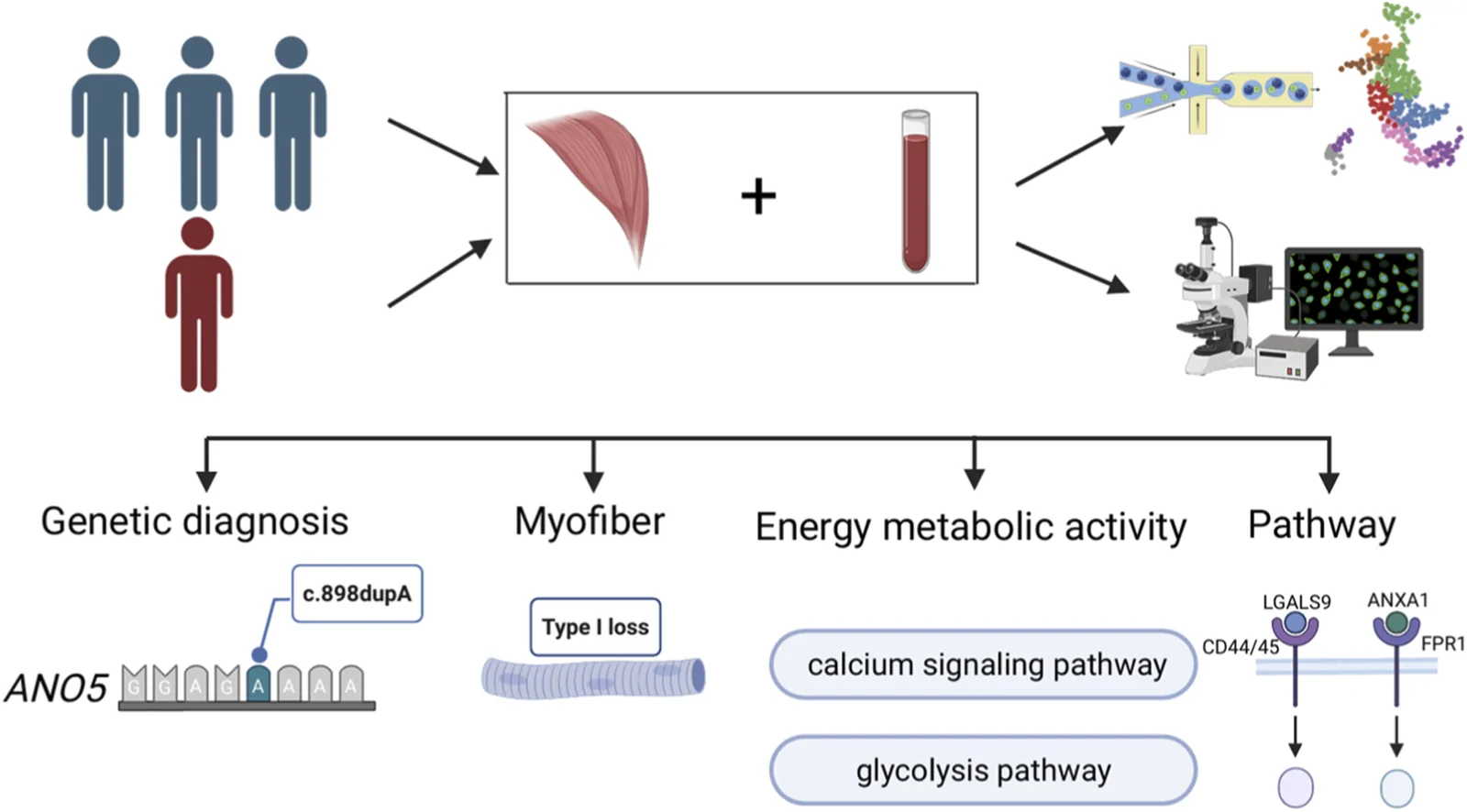

## 1 Introduction

Limb-girdle muscular dystrophy type R12 (LGMD R12, previously named LGMD 2L) is an autosomal recessive muscular dystrophy caused by pathogenic variants in *anoctamin-5 (ANO5)* (de Bruyn et al., 2023; D'Este et al., 2025; Marago et al., 2022). LGMD R12 belongs to a heterogeneous group of inherited neuromuscular disorders characterized by progressive proximal muscle weakness and atrophy, accompanied by changes in serum creatine kinase levels ranging from normal to markedly elevated (Kale et al., 2025). Clinically, it is characterized by progressive muscle weakness and atrophy predominantly affecting the pelvic and shoulder girdles, often leading to impaired mobility and functional decline in affected individuals (Angelini, 2020). The clinical features of this disease can complicate its differentiation from other types of limb-girdle muscular dystrophy and idiopathic inflammatory myopathies (IIMs) (Lorenzoni et al., 2023). Despite recent advancements in unraveling the genetic architecture of LGMD R12, effective therapeutic modalities remain elusive.

The *ANO5* gene, located on chromosome 11p14.3, encodes a transmembrane protein ANO5, belonging to the anoctamin family, which is primarily expressed in skeletal muscle, the heart, and the skin (Griffin et al., 2016; Mavillard et al., 2021). ANO5 is involved in multiple cellular processes, including calcium-activated chloride channel activity, plasma membrane repair, and muscle fiber homeostasis (Christiansen et al., 2022; Foltz et al., 2021). The intricate molecular cascades through which pathogenic variants in the *ANO5* gene disrupt muscle homeostasis and function remain incompletely delineated. This critical knowledge gap underscores the unmet need for comprehensive mechanistic investigations into LGMD pathogenesis.

Using single-cell RNA sequencing (scRNA-seq) analysis and experimental validation, we recently published a study on multiple subtypes of IIM (Wang et al., 2026). During the recruitment of IIM patients for that study, we identified a patient who was subsequently diagnosed with LGMD R12 through next-generation sequencing and Sanger sequencing.

To the best of our knowledge, this is the first single-cell transcriptome sequencing study of a patient with LGMD R12, and the analysis of muscle tissue (MUS) and peripheral blood from this patient has provided us with a comprehensive view of the individual’s pathological features.

## 2 Methods

### 2.1 Patient description

A 27-year-old male patient with no past medical history was admitted to the Department of Rheumatology, Tianjin Medical University General Hospital with a 9-month history of exercise-induced myalgia and hyperCKemia (3,079–8,386 U/L; a normal range 25–200 U/L). On physical examination, no rash was observed, muscle tone was normal, and muscle strength was grade V in the upper and lower limbs. Electromyography showed widespread myopathic changes in the symmetrical deltoid and gastrocnemius muscles. A biopsy of the right deltoid showed hypertrophy (Supplementary Figure 1A) and necrosis of muscle fibers (Supplementary Figure 1B), without evidence of inflammatory lymphocytic infiltration. Genetic analysis identified a homozygous pathogenic variant in exon 10 of the *ANO5* gene, c.898dupA (p.Ile300fs), confirming a diagnosis of LGMD R12. Meanwhile, we identified a heterozygous mutation in exon 10 of the *ANO5* gene, c.898dupA, in his parents. The patient received no specific treatment. At the 2-year follow-up, the patient remained capable of normal daily activities, with CK levels maintained at approximately 3,000-4,000 U/L.

### 2.2 scRNA-seq analysis

Skeletal muscle tissue was obtained from the patient and from 3 controls who underwent surgery for lipoma. Peripheral blood samples were obtained from the patient and three healthy controls (HCs). The HCs who fulfilled the following inclusion criteria were included: (1) normal blood test results, including blood count and liver and kidney function test results, and [2] no symptoms of autoimmune disease. The study was conducted in accordance with the Declaration of Helsinki. The study was approved by the hospital ethics committee of Tianjin Medical University General Hospital (approval number: IRB2021-YX-149-01), and written informed consent was obtained from the participants. Tissues were immediately processed to generate single-cell suspensions. PBMCs were isolated from peripheral blood samples using Ficoll–Paque PLUS density gradient centrifugation (GE Healthcare, United States). The PBMCs and muscle tissue were prepared for scRNA-seq library construction. Single-cell libraries were constructed using the SeekOne MM Single Cell 3’ Library Preparation Kit (SeekGene SO01V3.1, China) following the manufacturer’s instructions. The libraries were sequenced on an Illumina platform to generate 150-bp paired-end reads. The raw sequencing data were processed using the SeekSoul Tools pipeline (v1.2.1, SeekGene) with alignment to the human reference genome (GRCh38).

Data analysis was mainly performed in R (4.2.0). Seurat objects were generated using Seurat 4.4.0. Genes expressed in at least 3 cells and cells expressing at least 200 genes were retained for downstream analysis. Low-quality cells were removed if the number of expressed genes was <200 or >4,000 or if the proportion of mitochondrial gene expression was >20%. The cells were normalized using the NormalizeData function. Principal component analysis (PCA, dims 1:30) was performed, and batch effects were corrected using Harmony. Clusters were determined at a resolution of 1, and cell type annotations were adjusted accordingly. Cell types were defined based on differential gene expression, as determined using the Seurat FindAllMarkers function. Cell compositions were quantified based on the percentage of specific cell types within the samples. Nonlinear dimensionality reduction and visualization were performed using RunUMAP.

### 2.3 DEG analysis

Differentially expressed genes (DEGs) were identified separately for each cell type between the patient and HCs using the FindMarkers function in the Seurat package, with the parameters “min.pct” = 0.25 and “logfc.threshold” = 0.25.

### 2.4 ANO5 gene expression analysis

Nebulosa plots (Alquicira-Hernandez and Powell, 2021) provide a complementary visualization method to feature plots. We used Nebulosa plots to compute and visualize the density of *ANO5* expression across cell types. The density value reflects the local abundance of *ANO5*-expressing cells and their expression levels in the surrounding cells within the UMAP embedding.

### 2.5 Monocle analysis

Myocyte differentiation was analyzed using Monocle (version 2.26.0). Monocle orders single cells according to transcriptomic similarity and places them along a trajectory to infer developmental progression.

### 2.6 Gene ontology enrichment analysis

The ClusterProfile.v.4.6.2 R package’s EnrichGO function was used to analyze Gene Ontology (GO) terms. The three GO categories, namely, molecular function (MF), biological process (BP), and cellular component (CC), were all included in the analysis.

### 2.7 Immunofluorescence staining

Frozen biceps brachii sections were prepared, fixed, and subjected to antigen retrieval. Single- and dual-antibody staining using tyramide signal amplification were performed on frozen muscle sections. Briefly, after antigen retrieval, the sections were incubated in 5% bovine serum albumin (BSA), followed by incubation with antibodies against MYH7 (1:200, DSHB, BA-F8), MYH2(1:200, DSHB, SC-71), and Laminin (1:200, Sigma, L9393) overnight at 4 °C. The sections were then incubated with an HRP-conjugated secondary antibody (1:200, Servicebio, China) for 50 min and the corresponding tyramide (1:500, Servicebio, China) for 10 min at room temperature. The slides were counterstained with DAPI (1:10,000) for 5 min. Whole-slide imaging was performed using a Zeiss LSM 710 confocal microscope. One paraffin section from each of the healthy control and disease groups was stained, and the average fluorescence intensities of the MYH7 and MYH2 proteins were quantified using ImageJ software.

### 2.8 Cell communication analysis

Cell–cell interaction analysis was performed using CellChat v.1.6.1. All cell types were included in the analysis, with the Wilcoxon rank-sum test used at a significance level of 0.05, and ligand–receptor-mediated signaling interactions were then modeled using the law of mass action.

### 2.9 Single-cell metabolic pathway activity analysis

scMetabolism is an R package used to quantify metabolic activity at the single-cell level. Single-cell metabolic landscapes were generated to characterize metabolic signatures, calculate pathway activity, and analyze metabolic pathway heterogeneity (Xiao et al., 2019). Metabolic genes and pathways were obtained from the KEGG database. Pathway activity scores in these subtypes were calculated and visualized. Pathways with activity scores showing *p*-values < 0.01 were considered statistically significant.

### 2.10 Statistical analysis

Clinical samples in this study were collected from individuals independently. No statistical comparison was performed between the three healthy control datasets and the single LGMDR12 patient dataset in the manuscript.

Statistical analyses were conducted using R with the appropriate packages. DEGs were identified using volcano plots and Seurat’s Wilcoxon rank-sum test (single-cell level). GO enrichment was assessed using the hypergeometric test against the whole-genome background, and raw *p*-values were adjusted for multiple testing using the Benjamini–Hochberg FDR procedure.

## 3 Results

### 3.1 Single-cell transcriptome analysis reveals a decrease in myocytes in LGMD R12 muscle cells

This case in this study describes a young male patient (age 27) with identified pathogenic variants in the *ANO5* gene, leading to a final diagnosis of LGMD R12, characterized by exertional myalgia and hyperCKemia. Differential diagnoses include [1] inflammatory myopathy and [2] glycogen storage disease. With regard to inflammatory myopathy, we observed the young age of onset and the absence of characteristic symptoms (rash, arthralgia, and muscle weakness); however, negative findings on muscle biopsy make this diagnosis unlikely. With regard to glycogen storage disease, this diagnosis is not supported as the muscle biopsy revealed no vacuolar changes and the relevant genetic mutation was negative. Genetic analysis revealed that the patient was homozygous for a pathogenic variant in exon 10 of the *ANO5* gene (c.898dupA, p.Ile300fs), which has not been previously reported in other LGMD R12 cases (Bolduc et al., 2010). Furthermore, we identified a heterozygous mutation in exon 10 of the *ANO5* gene (c.898dupA) in his parents (Figures 1A,B). These results are consistent with the literature reports of asymptomatic hyperCKemia and exercise-induced myalgia observed in this type of myopathy (Palmio et al., 1993).

**FIGURE 1:**
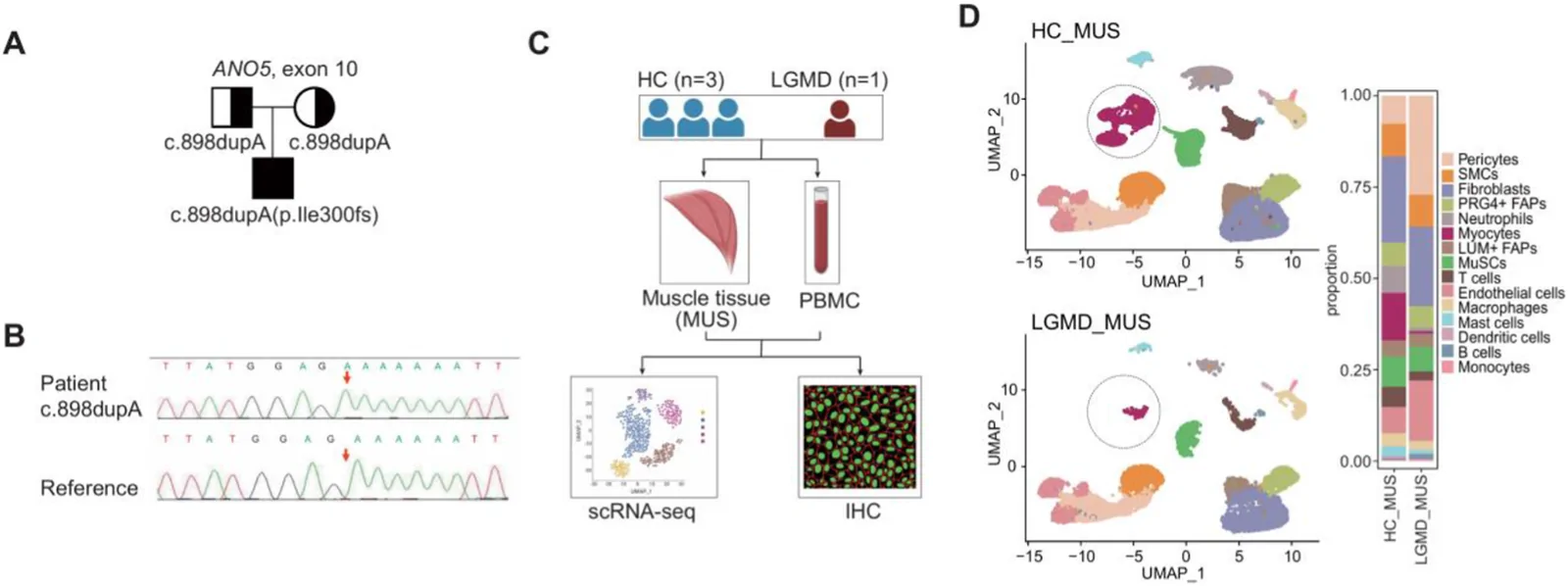
scRNA-seq profiling of MUS from the LGMD R12 patient and healthy controls. **(A)** Genetic variants of the *ANO5* gene in the patient and his parents. **(B)** Sanger sequencing results of the *ANO5* gene in the patient. **(C)** Overview of the experimental designs in the study. **(D)** Uniform manifold approximation and projection (UMAP) plot of 15 cell types (left) of muscle tissue. Relative percentages of cell types are shown on the right. MUS, biceps brachii muscle tissue.

To gain additional insights into this rare myopathy at such a young age, we performed single-cell sequencing of affected muscle and peripheral blood to elucidate its pathological molecular features. We believe that single-cell analysis of these two types of tissues is potentially more sensitive for dissecting pathological changes associated with the disease and may also reveal shared changes in the immunological landscape. The overall strategy of this study is presented in Figure 1C.

After quality control, a total of 38,477 muscle cells were included in the analysis, comprising 29,483 cells from 3 HCs and 8,994 cells from the LGMD R12 patient. Using classic gene expression markers (Supplementary Figure 2A), we identified 15 distinct clusters of muscle cells. As expected, the proportion of myocytes in the patient was significantly lower (Figure 1D). The significant reduction in myocytes in the LGMD R12 patient suggests a profound disruption in muscle homeostasis, emphasizing the need to investigate the drivers of myocyte loss. Consistent with the genetic diagnostic results, the expression of *ANO5* was barely detectable at the single-cell RNA level (Figure 2A). We prioritized and visualized DEGs between the patient and HCs in each cell type, which revealed widespread transcriptomic perturbations (Supplementary Figure 2B). Furthermore, we reclustered myocytes from the entire UMAP dataset. Myocyte subclusters were characterized based on the expression of *PAX7*, *DLK1*, *MYF5*, *MYH2*, *ATP2A1*, *TNNT3*, *TNNT1*, *MYH7*, *ATP2A2*, *ACTC1*, and *MYH8* (Supplementary Figure 2C). We identified four subtypes of myofibers, namely, muscle progenitor cells (MPCs), type I (slow-twitch), type II (fast-twitch), and an intermediate state transitioning from MPCs to myofibers (‘MPCs-to-Type II’ state) (Kedlian et al., 2024) (Figure 2B). Notably, the observed increase in MPCs in the subject, coupled with the absence of type I myofibers, suggests impaired differentiation of MPCs, which fail to develop into mature myofibers.

**FIGURE 2:**
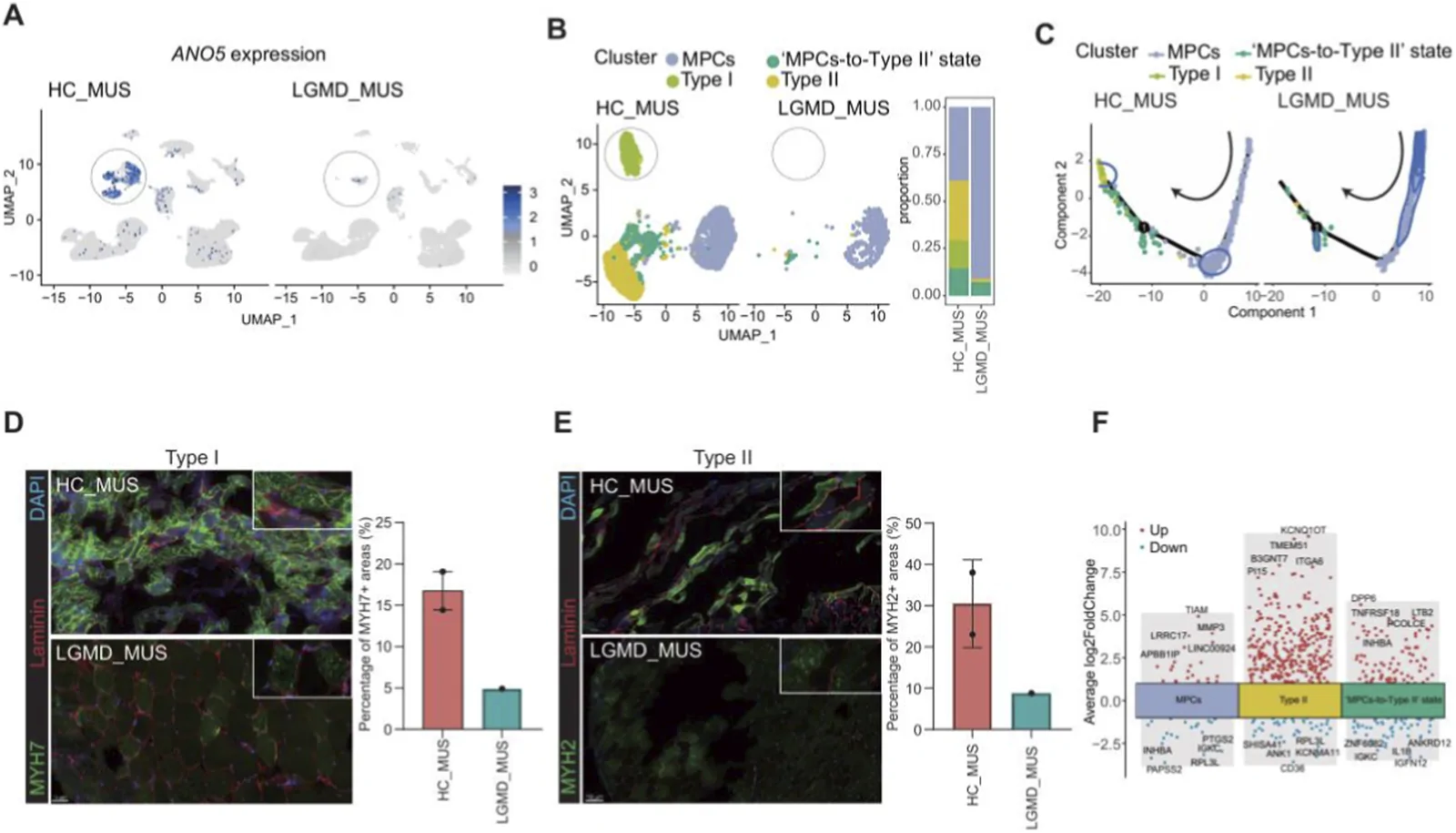
Analysis of muscle cells from the observed LGMD R12 patient and healthy controls. **(A)** Expression of *ANO5* in muscle cells from the LGMD R12 patient and healthy controls. **(B)** UMAP plots displaying four subtypes of myocytes (left). Bar plot showing the cluster abundance (right). **(C)** Developmental trajectories of myocytes. **(D,E)** Representative multiplex staining of Laminin (red), MYH7 (green in D), MYH2 (green in E), and DAPI (blue) (left). Statistics for MYH7^+^ and MYH2^+^ cells (right). **(F)** Differentially expressed genes (DEGs) in each cell type.

To further explore the development of LGMD R12 muscle cells, we performed a Monocle analysis, and the results indicated that the patient’s muscle cells largely remained in an undifferentiated state (Figure 2C). Immunofluorescence staining also confirmed that the MYH7^+^ (type I) and MYH2^+^ (type II) myofibers were reduced in this patient with LGMD R12 compared with HCs (Figures 2D,E). A multi-component volcano plot illustrated that RPL3L, which is involved in muscle contraction pathways in myocytes (Lin et al., 2022; Miller et al., 2023), exhibited reduced expression (Figure 2F).

Interestingly, the results of GO analyses showed that ‘leukocyte migration’ and ‘cell chemotaxis’ BPs were highly enriched in LGMD R12 (Figure 3A). The most significantly downregulated BPs in LGMD R12 were ‘muscle system process’ and ‘striated muscle cell differentiation,’ which indicate muscle atrophy and developmental disorders (Figure 3B). Of note, our pathway analyses revealed a notable decrease in the activity of metabolic pathways in our LGMD R12 patient, involving nucleotide metabolism, energy metabolism, carbohydrate metabolism, and amino acid metabolism (Figure 3C). This metabolic reprogramming is critical as it suggests a shift toward a maladaptive energy utilization strategy, exacerbating muscle degeneration. A previous study has demonstrated that ANO5 protein deficiency impairs the plasma membrane repair function of myoblasts (Chandra et al., 2019). Therefore, these dysregulated energy and biosynthetic metabolic processes (particularly amino acid and carbohydrate pathways) in muscle cell subpopulations may contribute to the contractile dysfunction observed in LGMD R12.

**FIGURE 3:**
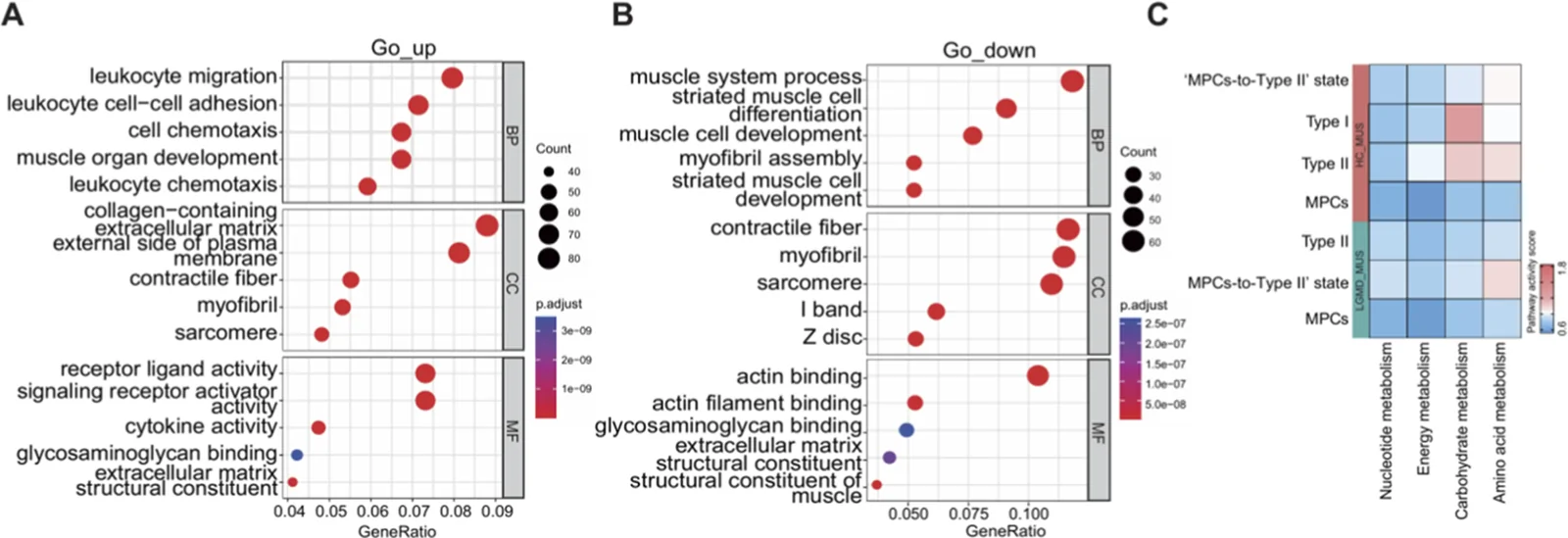
GO analysis and scMetabolism analyses of muscle cells from the LGMD R12 patient and healthy controls. **(A,B)** Biological process enrichment analysis (Gene Ontology pathways) in myocytes compared with HCs. **(C)** Heatmap showing metabolic pathway activity inferred by scMetabolism in muscle cells.

Furthermore, the fiber type-specific loss of energy metabolism in type I fibers further suggests a metabolic shift from oxidative phosphorylation to glycolysis—a maladaptive response that could exacerbate muscle atrophy. We performed Kyoto Encyclopedia of Genes and Genomes (KEGG) pathway enrichment analyses and found that the calcium signaling pathway and glycolysis were dysregulated (Supplementary Figures 2D-E). Transcriptome sequencing data from patients with LGMD R12 suggest that glycolytic pathway activity and cellular metabolic activity are significantly downregulated (Depuydt et al., 2022). Moreover, this study revealed that gene sets associated with energy metabolism, glycolysis, oxidative phosphorylation (OXPHOS), and protein metabolism were significantly downregulated, which is consistent with our finding that glycolytic pathway activity and cellular metabolic activity were also significantly downregulated. These dynamics reflect disrupted cellular signaling and metabolic reprogramming, underscoring a pathological progression in LGMD R12.

### 3.2 The LGALS9–CD45/CD44 and ANXA1–FPR1 pathways are activated in muscle lesions and PBMCs

To gain insight into the signaling molecules underlying immune cell-mediated inflammation, we extracted immune cells, including mast cells, neutrophils, T cells, macrophages, dendritic cells, B cells, and monocytes, from muscle tissue based on the UMAP plot. These cells were then subjected to dimensionality reduction and reclustering, yielding six cell types based on canonical gene expression markers (Figure 4A; Supplementary Figure 3A). Compared with controls, the patient showed a significant increase in the proportion of B cells in muscle tissue (Figures 4B,C). Similar to the findings in muscle cells, GO enrichment analysis of immune cells in muscle tissue indicated downregulation of pathways related to muscle tissue development and upregulation of immunological pathways (Figures 4D,E). Additionally, pathways related to muscle ultrastructural components, including myofibrils and sarcomeres, were downregulated, with marked reductions in actin-binding and glycosaminoglycan-binding activities. These results indicate that this patient is characterized by impaired muscle cell development and enhanced leukocyte migration and chemotaxis. We used CellChat to investigate putative interactions among immune cells in the patient (Figures 5A,B). Compared with HCs, the LGALS9–CD45/CD44, IL1β–IL1R2, CXCL8–CXCR2/1, CXCL3–CXCR2/1, CXCL2–CXCR2/1, and ANXA1–FPR1 pathways between macrophages and neutrophils were enriched in the patient (Figure 5C).

**FIGURE 4:**
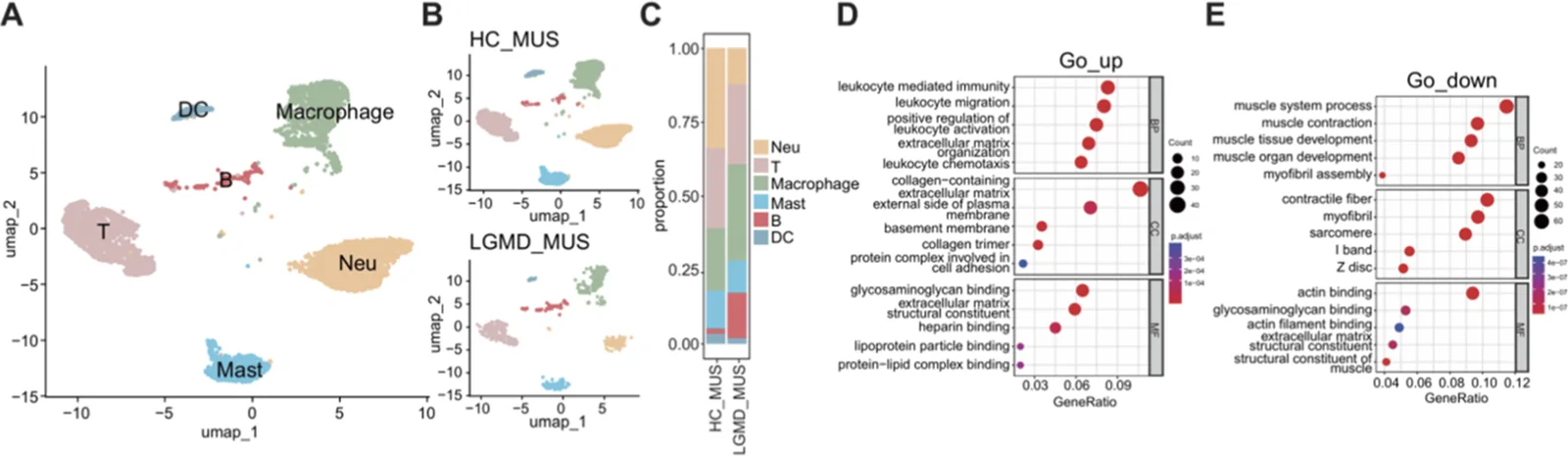
Single-cell atlas of immune cells in muscle cells from the patient and healthy controls. **(A)** UMAP plot showing immune cells in muscle tissue. **(B)** UMAP plot showing the six cell subtypes from the patient and HCs. **(C)** Proportions of immune cells in muscle cells. **(D)** Upregulated GO pathways in the indicated immune cells. **(E)** Downregulation of GO pathways of immune cells in muscle cells.

**FIGURE 5:**
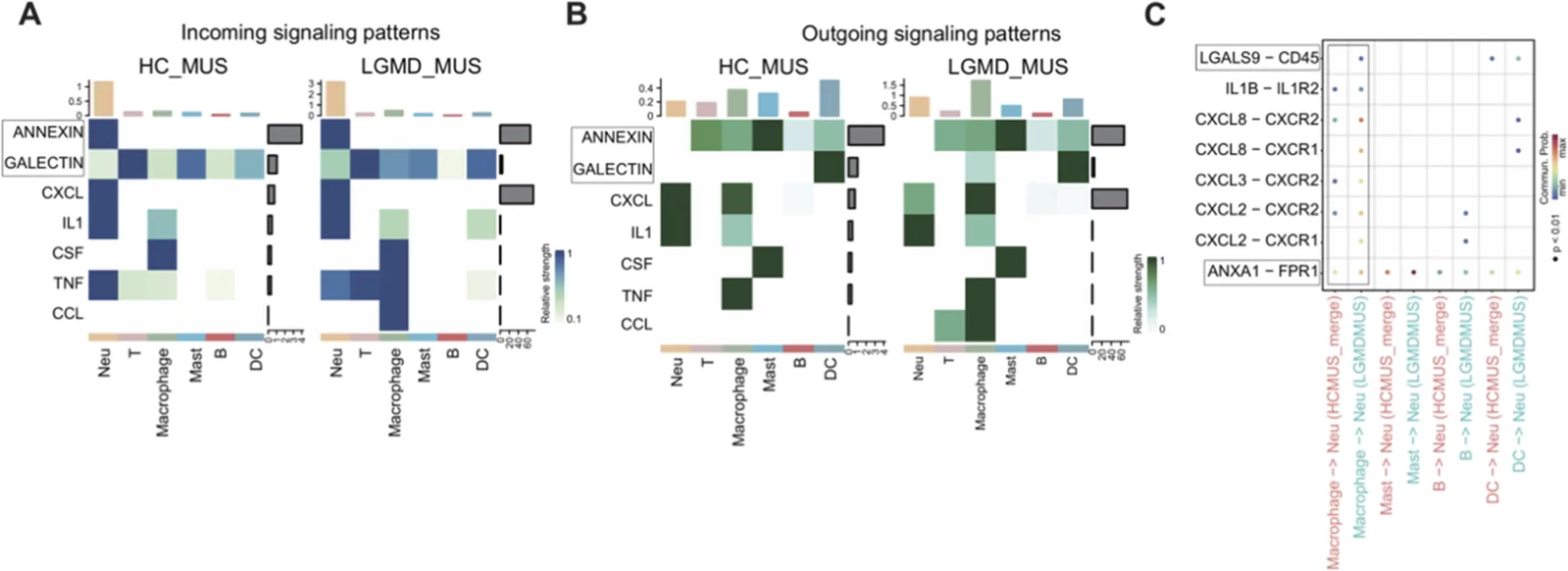
Analysis of ligand–receptor interactions and cellular communication. The cell–cell communication analysis is based on the scRNA-seq datasets of immune cells from the muscles of the patient and HCs. **(A)** Predominant incoming signaling patterns. **(B)** Predominant outgoing signaling patterns. **(C)** Dot plot showing the significant ligand–receptor pairs across immune cells in muscle cells.

To further explore inflammatory signaling pathways involving immune cells, we analyzed PBMCs from our LGMD R12 patient. Based on the expression levels of the classic markers, *LEF1*, *CCR7*, *TCF7*, *CTLA4*, *CD40LG*, *CD8A*, *GNLY*, *PRF1*, *NKG7*, *MYOM2*, *SPON2*, *CD14*, *VCAN*, *CD163*, *MS4A1*, *CD22*, *MS4A7*, *CSF1R*, *MSR1*, *CD1C*, *MRC1*, *LTF*, *DEFA3*, *CAMP*, *TCF4*, *IRF8*, *LILRA4*, *PPBP*, *PF4*, *SPINK2*, *CD34*, *CSF2RB*, *GATA2*, *MS4A2*, *IGHG1*, *JCHAIN*, and *XBP1* (Supplementary Figure 3B), we analyzed 8,161 PBMCs from the LGMD R12 patient and 25,685 PBMCs from three healthy controls, which clustered into 14 major cell types (Figures 6A,B). Strikingly, an increased number of B cells was also observed in PBMCs (Figure 6C), mirroring the findings observed in immune cells from muscle tissue. Integrated GO enrichment analysis revealed the upregulation of pathways involved in immune regulation (Figure 6D), while the downregulated pathways were involved in the response to lipopolysaccharide and vesicle lumen (Figure 6E).

**FIGURE 6:**
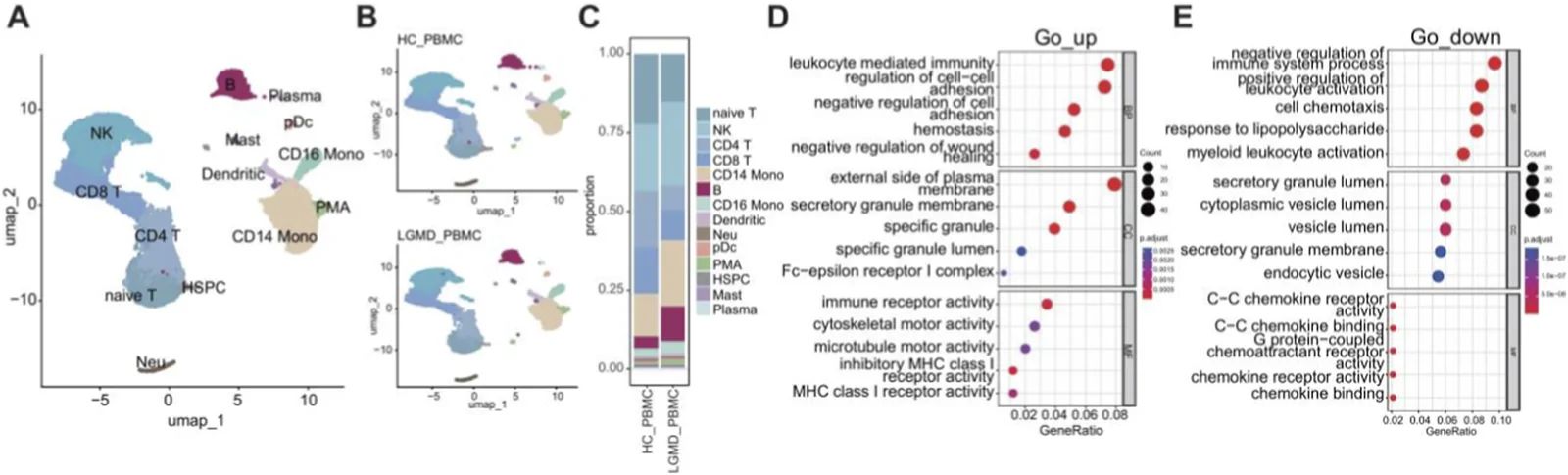
Single-cell atlas of immune cells in the peripheral blood from the patient and healthy controls. **(A–C)** UMAP plot displaying 14 cell clusters in PBMCs. The proportions of these cell clusters in PBMCs are shown in **(C)**. **(D)** Upregulated GO pathways in PBMCs compared with HCs. **(E)** Downregulated GO pathways in PBMCs.

Interestingly, the ANNEXIN and GALECTIN signaling pathways were also active in PBMCs (Figures 7A,B). We found that the NAMPT–(ITGA5 + ITGB1), IL16–CD4, GRN–SORT1, and CCL5–CCR1 pathways were active in the patient, in addition to the LGALS9–CD45/CD44 and ANXA1–FPR1 pathways (Figure 7C). Therefore, the LGALS9–CD45/CD44 and ANXA1–FPR1 pathways may converge as critical pathogenic nodes in the patient.

**FIGURE 7:**
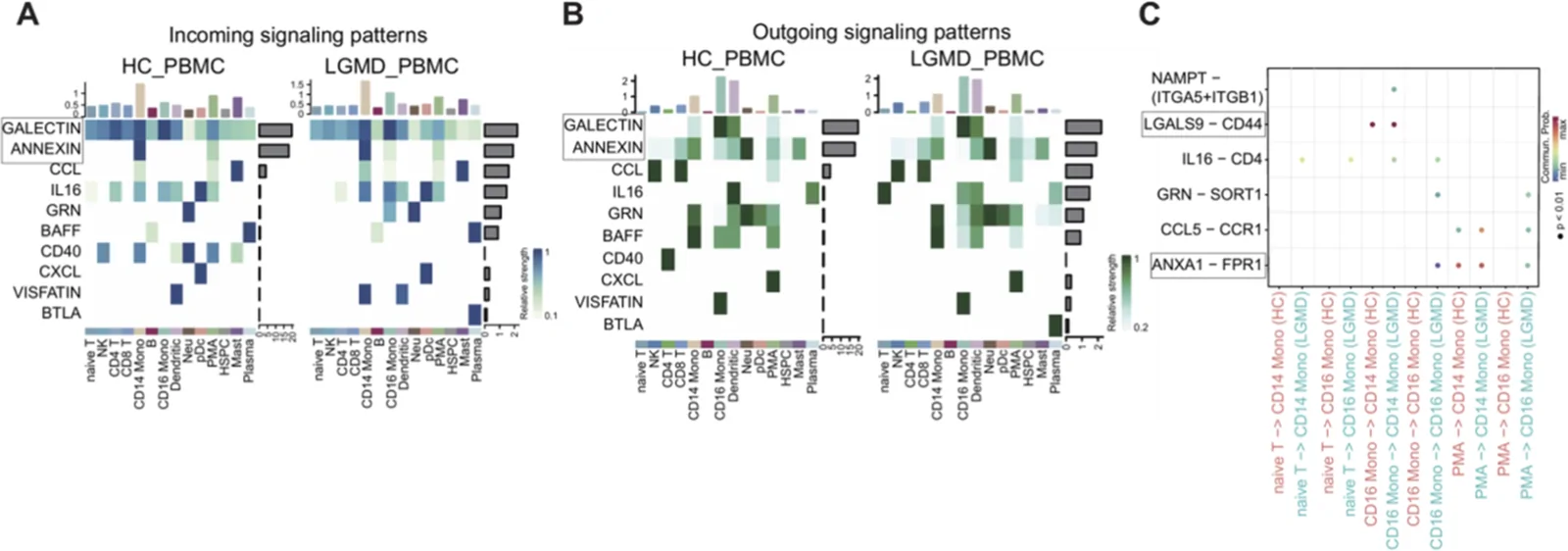
Analysis of ligand–receptor interactions and cellular communication in the scRNA-seq datasets of PBMCs. **(A)** Predominant incoming signaling patterns. **(B)** Predominant outgoing signaling patterns. **(C)** Dot plot showing significant ligand–receptor pairs mediating cellular communication among cell types in PBMCs.

## 4 Discussion

In this study, we detailed, for the first time, the single-cell transcriptome profiles of muscle and peripheral blood samples from an LGMD R12 patient. The observed increase in muscle progenitor cells (MPCs) in muscle tissue, along with a deficiency of type I myofibers, suggests a pivotal disruption in muscle development and differentiation, which warrants further exploration in animal models. The muscle weakness in the patient may be attributed to impaired differentiation of MPCs, which fail to properly differentiate into mature myofibers.

In the diseased sample, the proportion of MPCs in the muscle tissue lesions increased significantly, while the proportions of type I and type II muscle fibers decreased significantly, which may be indicative of muscle weakness. Furthermore, immunofluorescence staining revealed a limited presence of both type I and type II muscle fibers. We used laminin to mark the outlines of muscle fibers (Kedlian et al., 2024). As a component of the basement membrane, laminin has an indirect relationship with muscle cells. The apparent contradiction between the absence of lymphocyte infiltration in the patient’s muscle biopsy and the single-cell RNA sequencing data may be attributed to two factors. First, the muscle tissue obtained via biopsy was limited in sampling area, which may have failed to detect immune cells. Second, electromyography results revealed mild neurogenic damage in the left gastrocnemius muscle, and such mild injury may have triggered the activation of inflammatory signaling pathways.

One limitation of our study is that we did not verify the activation of the LGALS9–CD45/CD44 and ANXA1–FPR1 pathways. Due to the lack of animal and cell models, we did not assess changes in the key molecules of these two signaling pathways. As described in Results Section 3.1, with disease progression (reflected by elevated Mercuri scores), muscle-specific metabolic pathways, including glycolysis and mitochondrial aerobic respiration, exhibited progressive downregulation (Depuydt et al., 2022). This finding is consistent with our conclusion that glycolytic pathway activity and cellular metabolic function were markedly suppressed. In the muscle tissue of this patient, expression levels of immune cell infiltration-related genes (e.g., *SPP1*, *IGHG1/2*, *SAA1*, and *LTK*) were significantly upregulated, and gene sets associated with immune responses and phagocytosis were markedly enriched. These findings demonstrate that inflammation-associated signaling pathways are activated in the LGMD R12 patient.

In conclusion, we used single-cell transcriptome technology for the first time to describe the characteristics of muscle lesions and peripheral blood in an LGMD R12 patient. Further studies are needed to determine the causes of myofiber loss and validate implicated signaling pathways.

## What this study ADDS

1. Genetic testing identified a homozygous pathogenic variant in exon 10 of *ANO5*, c.898dupA (p.Ile300fs), confirming a diagnosis of LGMD R12.
2. Using single-cell RNA sequencing, we constructed high-resolution single-cell transcriptional atlases of both biceps brachii muscle tissue (MUS) and peripheral blood mononuclear cells (PBMCs) derived from an LGMD R12 patient.
3. Our results revealed a marked reduction in MYH7^+^ and MYH2^+^ myofibers in an LGMD R12 patient.
4. We observed reduced energy metabolic activity in muscle cells, accompanied by the downregulation of genes involved in both the calcium signaling and glycolytic pathways in this LGMD R12 patient.
5. We identified activation of the LGALS9–CD45/CD44 and ANXA1–FPR1 pathways in both MUS and PBMCs from the described LGMD R12 patient.

## How this study might affect research, practice, or policy

This study is the first to reveal cellular and molecular changes in LGMD R12 at single-cell resolution. The near-complete absence of MYH7^+^ and MYH2^+^ muscle fibers was validated by immunofluorescence staining. Additionally, we observed synergistic activation of the LGALS9–CD45/CD44 and ANXA1–FPR1 pathways in both muscle lesions and PBMCs. These findings offer insights into the etiology and potential therapeutic strategies for this recessive genetic myopathy in clinical management.

## Data Availability

Data are available in a public, open access repository. The raw sequence data reported in this paper have been deposited in China National Center for Bioinformation/Beijing Institute of Genomics, Chinese Academy of Sciences (GSA-Human: HRA028255) that are publicly accessible at: https://ngdc.cncb.ac.cn/gsa-human. The R scripts used for the analyses in this study have been deposited in a public GitHub repository. The file named LGMDR12MUS1vs3 corresponds to the single-cell transcriptional analysis of biceps brachii muscle tissue (MUS) from one LGMD R12 patient and three healthy donors. The file named LGMD_01 corresponds to the single-cell transcriptional analysis of peripheral blood mononuclear cells (PBMCs) from one LGMD R12 patient and three healthy controls. These scripts are available at: https://github.com/cailab-bio/LGMD_code. Any additional information required to reanalyze the data reported in this paper is available from the corresponding author contact upon request.
