# Supplementary material for: Single-cell transcriptome analysis of muscle and blood tissues from a patient with a rare homozygous ANO5 variant causing limb-girdle muscular dystrophy type R12
Source: Front Genet. 2026 Oct 19;17:1833196. doi: 10.3389/fgene.2026.1833196 (PMC13724551; doi:10.3389/fgene.2026.1833196)
Supplement: Supplementary file 1 [file DataSheet1.docx]

**Supplementary information**

Additional file: Supplementary

Figure S1. HE staining (Hematoxylin-Eosin staining) of the biceps brachii biopsy specimen.

(A) Muscle fiber hypertrophy (yellow arrows) (200-fold magnification).

(B) Necrosis of muscle cells with phagocytosis (blue circle) (200-fold magnification).

Figure S2. Related to Figure 1.

(A) Dot plots showing the expression of representative marker genes. The dot size indicates the percentage of cells expressing the indicated gene, and the color gradient reflects the average expression level. MuSCs: muscle stem cells; SMCs: smooth muscle cells.

(B) Volcano plot displaying DEGs in each cell type in muscle cells between the LGMD R12 patient and HCs.

(C) Dot plots showing marker genes in MPCs (myogenic progenitor cells), Type I, ‘MPCs-to-Type II’ intermediate state and Type II.

(D) GSEA (Gene Set Enrichment Analysis) showing the enrichment characteristics of the calcium signaling pathway in the ranking of differentially expressed genes.

(E) GSEA showing the glycolysis pathway in the ranking of differentially expressed genes.

Figure S3. Related to Figure 2.

(A) Bubble plots illustrating the markers of different immune cells in muscle cells. DC: dendritic cell; B: B cell; Mast: mast cell; Macrophage: macrophage; T: T cell; Neu: neutrophil.

(B) Bubble plots illustrating the markers of different immune cells in PBMCs. Plasma: plasma cell; Mast: mast cell; HSPC: hematopoietic stem and progenitor cell; PMA: platelet-monocyte aggregates; pDC: plasmacytoid dendritic cell; Neu: neutrophil; Dendritic: dendritic cell; CD16 Mono: CD16 monocyte; B: B cell; CD14 Mono: CD14 monocyte; CD8 T: CD8 T cell; CD4 T: CD4 T cell; NK: natural killer cell; naïve T: naïve T cell.
